# Does age matter? Acute appendicitis in octogenarians vs. younger patients - results of a German multicenter analysis

**DOI:** 10.1007/s00423-026-04202-3

**Published:** 2026-09-08

**Authors:** Jasmin M. M. Schmitt, Nina Kühler, Sebastian Schaaf, Arnulf G. Willms, Johan F. Lock

**Affiliations:** 1 Department of General, Visceral, Transplant, Vascular and Pediatric Surgery, University Hospital Wuerzburg, Wuerzburg, Germany; 2 Department of General, Visceral and Thoracic Surgery, Bundeswehr Central Hospital Koblenz, Koblenz, Germany

**Keywords:** Appendicitis, Emergency surgery, Acute surgery, Octogenarian, Multicenter study

## Abstract

**Background:**

Acute appendicitis is a common indication for emergency abdominal surgery. Octogenarians (≥ 80 years) are considered high risk due to comorbidities and reduced physiological reserve. This study examined age-related differences in symptoms, surgical management, and outcomes in patients undergoing surgery for acute appendicitis.

**Methods:**

This multicenter retrospective cohort study includes 1,582 surgically treated patients from 41 German hospitals, comprising 1,533 non-octogenarians (18–79 years) and 49 octogenarians (≥ 80 years).

**Results:**

Octogenarians had higher comorbidity burden and ASA classification (both *p* < 0.001). Clinical presentation was largely comparable; however, octogenarians experienced lower pain intensity (*p* = 0.05) and had higher CRP levels (*p* < 0.001). Complicated appendicitis was more prevalent in octogenarians (63.3% vs. 28.1%; *p* < 0.001), with higher rates of perforation, peritonitis, and abscess formation (all *p* < 0.001). Consequently, extended resections (*p* = 0.01), open or converted surgery (*p* < 0.001), drainage (*p* < 0.001), and stoma formation (*p* = 0.01) were more common. Postoperative complications (*p* < 0.001), mortality (*p* = 0.002), ICU admission (*p* < 0.001), duration of antimicrobial therapy (*p* < 0.001), and hospital stay (*p* < 0.001) were all increased. Following propensity score matching, outcomes were largely comparable between octogenarians and younger patients, indicating the comorbidity spectrum of the elderly, rather than chronological age alone, as key determinants.

**Conclusion:**

Despite a similar initial presentation, octogenarians present with more advanced appendicitis and significantly worse postoperative outcomes. This emphasizes the need for age- and comorbidity-adapted diagnostic and perioperative strategies.

**Supplementary Information:**

The online version contains supplementary material available at https://doi.org/10.1007/s00423-026-04202-3.

## Introduction

Acute appendicitis is among the most common reasons of emergency abdominal surgery worldwide and affects patients of all ages [1, 2].

While the disease course is generally well characterized, elderly patients, particularly those aged 80 years and older (octogenarians), are often thought to present with atypical symptoms [3]. Comorbidities, frailty, and age-related physiological changes also increase perioperative risk in this group [4–6]. Overall, the elderly experience higher morbidity and mortality than younger patients, especially in acute surgeries [7–9].

The distinction between uncomplicated and complicated appendicitis is crucial for treatment planning, as it informs decisions regarding operative versus non-operative management [1, 2]. Uncomplicated appendicitis is defined as phlegmonous inflammation without perforation, abscess formation, or peritonitis. In contrast, complicated appendicitis encompasses gangrenous or ulcero-phlegmonous appendicitis, as well as perforation, peritonitis, or abscess [1].

Scoring systems, such as the modified Alvarado score (MASS), and isolated physical examination signs, such as McBurney’s, Blumberg’s, and Lanz’s signs, are widely used to support the diagnosis of appendicitis [1, 2, 10]. Laboratory markers, particularly C-reactive protein (CRP), may provide valuable adjunctive information to guide timely diagnosis and treatment [11].

Given the aging population [12] and the rising proportion of octogenarians requiring surgical care [13], there is a pressing need to understand age-related differences in clinical presentation, intraoperative findings, and postoperative outcomes [5]. A substantial proportion of clinical evidence and guidelines is derived from randomized trials that systematically exclude elderly patients, particularly those with multiple comorbidities [14].

A detailed characterization of these differences may inform risk-adapted surgical strategies and optimize perioperative management in elderly patients with appendicitis [15]. Using an extensive multicenter dataset, we aimed to compare the clinical presentation, surgical management and postoperative outcomes of non-octogenarians and octogenarians undergoing surgery for acute appendicitis.

## Methods

The data collection was conducted as previously reported [16]. A total of forty-one surgical departments participated and submitted their data by August 20, 2020. Details regarding the care level and numbers of cases of the participating hospitals is summarized in Table 1.

### Study design

The study was designed as a multicenter retrospective cohort study assessing cases of appendicitis during the COVID-19 lockdown and comparing them with a control cohort from the preceding year. The study protocol was approved by the ethics committees of the administrative study centers of Würzburg (Ethikkommission der Universität Würzburg, No. 20200702 01) and Koblenz (Landesärztekammer Rheinland-Pfalz No.: 2020–15159). In addition, several participating centers obtained approval from their responsible local review boards in accordance with local requirements. Due to its retrospective design, registration in an ICMJE-compliant primary registry was not performed.

### Inclusion and exclusion criteria

The study included patients (aged ≥ 18 years) diagnosed with acute appendicitis (ICD-10 K35, K37) who received operative treatment. Individuals with chronic appendicitis or other diseases of the appendix (ICD-10 K36, K38) were excluded, as were patients undergoing simultaneous appendectomy during abdominal surgery for unrelated indications. Cases lacking data concerning age, the surgical therapy or the histopathological results were not considered for analysis. A total of 333 patients were therefore excluded from further analysis. Octogenarians were defined as patients aged ≥ 80 years, and all remaining patients (aged 18–79 years) were considered controls.

**Fig. 1:**
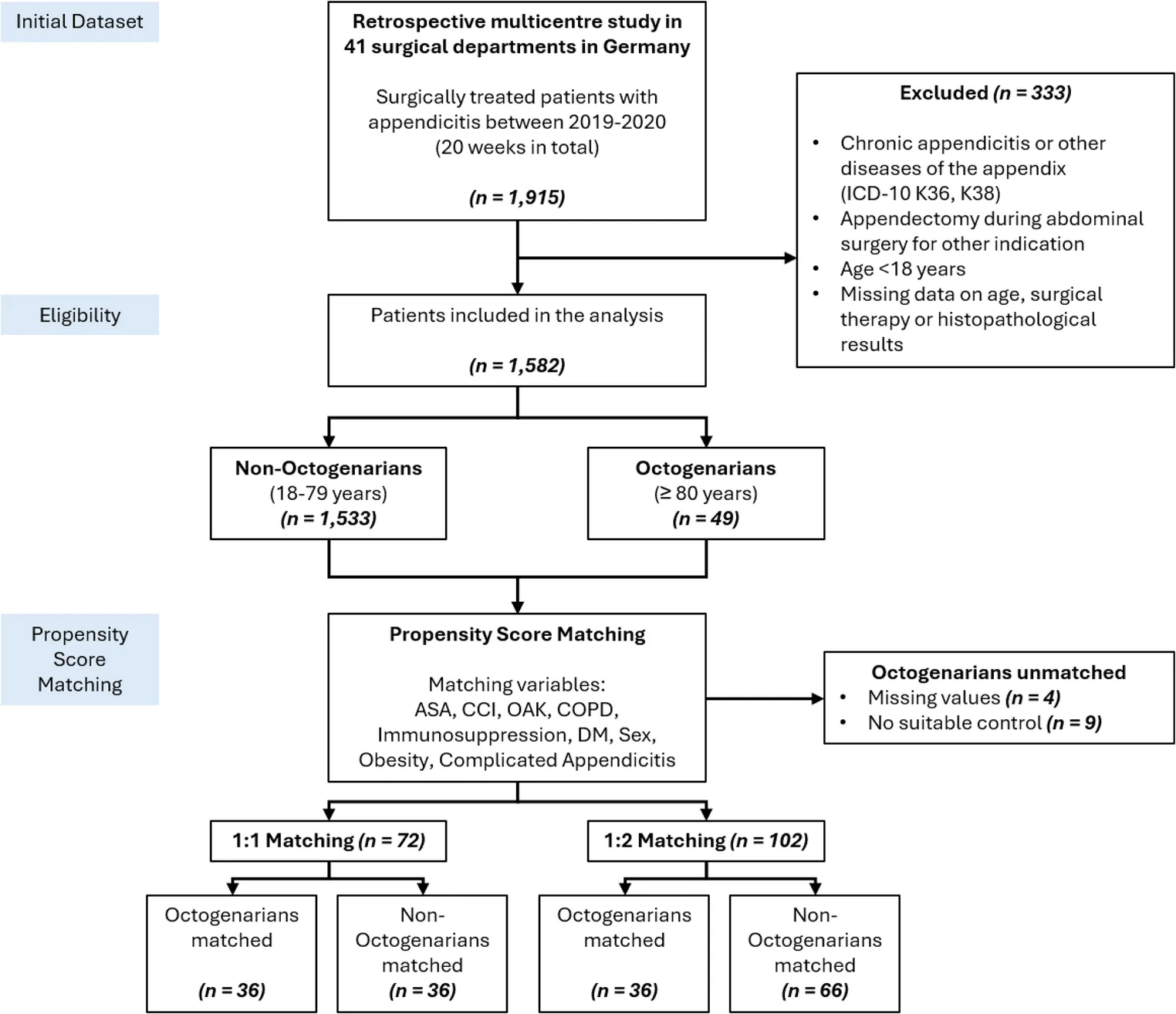
Flow diagram of case selection and propensity score matching. Abbreviations: ASA, American Society of Anesthesiologists Score, CCI, Charlson comorbidity index; COPD, chronic obstructive pulmonary disease; DM, Diabetes mellitus; OAK, Oral anticoagulation

### Collection of data

The CAMIN (Surgical Working Group for Military and Emergency Surgery) Study Group of the German Society for General and Visceral Surgery (DGAV) compiled an anonymized database (Excel 2016, Microsoft, USA), ensuring patient confidentiality. Perioperative variables were collected from hospital information systems, internally validated, and subsequently transferred to the study database. All variables were checked in detail for plausibility. Plausible entry errors were corrected, and implausible values were deleted. The database comprised patient demographics (age, sex) and comorbidities, including the American Society of Anesthesiologists ASA score [17] and the Charlson comorbidity index [18]. Symptom presentation and preoperative assessment were recorded, including the duration of symptoms, clinical signs (Lanz, Blumberg, McBurney), laboratory inflammatory markers (leukocyte count and CRP), and modified Alvarado score (MASS) [10]). Intraoperative findings encompassed the presence of peritonitis, perforation, abscess formation, and the Mannheimer Peritonitis Index [19]. Additionally, the histopathological findings of appendicitis were documented. Postoperative outcome included length of stay (LOS), intensive-care-unit (ICU) utilization, mortality, and postoperative complications graded according to Clavien-Dindo classification [20].

### Statistical analysis

Data were imported to JASP, Version 0.95.4, and Python (Python Software Foundation, Wilmington, DE, USA) with the libraries *pandas*, *scikit-learn*, *SciPy*, and *statsmodels* for statistical analysis. Variables were converted to numeric format where necessary. Normality and homogeneity of variances were checked using the Shapiro-Wilk and Levene’s test, respectively. Continuous variables are (due to non-normality) provided as median with interquartile range (IQR) and categorical variables as counts with percentages. Continuous variables were compared using the Mann–Whitney U test. For normally distributed data, Student’s t-test (or Welch’s t-test where appropriate) was applied. Categorical variables were compared using the chi-squared test. P values less than 0.05 were considered statistically significant. Octogenarians were defined as patients aged ≥ 80 years, and all remaining patients were considered controls.

### Propensity score matching

Propensity score matching was performed to reduce selection bias and achieve balanced comparability between octogenarians and younger patients. The matching model included sex, ASA score, Charlson Comorbidity Index, diabetes mellitus, COPD, oral anticoagulation, immunosuppression, and obesity. To account for the higher rate of complicated appendicitis in Octogenarians we also included appendicitis severity (complicated vs. uncomplicated appendicitis) as a key covariate.

### Propensity score estimation and matching procedure

Propensity scores were estimated using logistic regression, with age ≥ 80 years as the dependent variable and the predefined baseline covariates as independent variables. Scores represent the probability of belonging to the octogenarian group conditional on baseline characteristics. Nearest-neighbor matching without replacement was applied using a caliper width of 0.2 standard deviations of the logit of the propensity score. Primary matching was performed in a 1:1 ratio, yielding 36 matched pairs. As a sensitivity analysis, 1:2 matching (one octogenarian matched to two controls) was conducted using the same algorithm and caliper. Covariate balance before and after matching was assessed using standardized mean differences (SMD). Patients with missing values (Octogenarians: 4 cases, Non-Octogenarians: 255 cases) in any of these covariates were excluded (complete-case analysis).

## Results

A total of 1,582 patients were included in the analysis, comprising 1,533 non-octogenarians (aged 18–79 years) and 49 octogenarians (aged ≥ 80 years), see Fig. 1. There were no significant differences in the distribution of sex (*p* = 0.17), BMI (*p* = 0.82), or the prevalence of obesity (*p* = 0.10) between the two groups. Octogenarians exhibited a significantly higher comorbidity burden (CCI; *p* < 0.001), with fewer patients without comorbidities (30.4% vs. 82.9%), and a markedly higher proportion with major comorbidities (CCI ≥ 5: 32.6% vs. 1.7%). Accordingly, COPD (10.2% vs. 1.2%; *p* < 0.001), immunosuppression (8.2% vs. 1.3%; *p* < 0.001), and oral anticoagulant use (32.7% vs. 2.0%; *p* < 0.001) were more prevalent in octogenarians. However, the prevalence of diabetes mellitus did not differ (*p* = 0.11).

The ASA score was also significantly higher in octogenarians (*p* < 0.001), with the majority classified as ASA 3–4 (61.2% vs. 8.9%). Most patients received treatment in secondary care hospitals (44.9% vs. 39.4%), followed by university hospitals (28.6% vs. 29.9%). Tertiary hospitals treated 12.2% of octogenarians and 18.9% of non-octogenarians, while primary care hospitals treated 14.3% vs. 11.8%, respectively. Overall, the distribution of treating hospital levels was comparable between age groups (*p* = 0.63). For a complete overview, refer to Table 1 and 2.

**Table 1:** Level of hospital care and number of patients

	No. (%)			P value
Level	Hospitals	Non-Octogenarians (age 18–79)	Octogenarians (age ≥ 80)	
University hospital	13 (31.7)	459 (29.9)	14 (28.6)	0.63
Tertiary	6 (14.6)	289 (18.9)	6 (12.2)	
Secondary	16 (39)	604 (39.4)	22 (44.9)	
Primary	6 (14.6)	181 (11.8)	7 (14.3)	

**Table 2:** Patient characteristics

Variable	No. (%)		*P* value
	Non-Octogenarians (age 18–79)	Octogenarians (age ≥ 80)	
n =	1533	49	**< 0.001**
Sex; m:f	754/779	29/20	0.17
Age, y, *median (IQR)*	35 (25)	83 (5.0)	**< 0.001**
BMI, *median (IQR)*	25.0 (6.3)	24.9 (5.0)	0.82
Obesity, BMI > 30 kg/m^2^	252 (16.4)	4 (8.2)	0.10
Comorbidity^1^			
None, CCI 0	1215 (82.9)	14 (30.4)	**< 0.001**
Minor, CCI 1–2	174 (11.9)	12 (26.1)	
Moderate, CCI 3–4	52 (3.5)	5 (10.9)	
Major, CCI ≥ 5	25 (1.7)	15 (32.6)	
Diabetes	57 (3.7)	4 (8.2)	0.11
COPD	19 (1.2)	5 (10.2)	**< 0.001**
Immunosuppression	20 (1.3)	4 (8.2)	**< 0.001**
Oral Anticoagulation	31 (2.0)	16 (32.7)	**< 0.001**
ASA score			
1	727 (47.4)	1 (2.0)	**< 0.001**
2	630 (41.1)	15 (30.6)	
3	123 (8.0)	27 (55.1)	
4	13 (0.9)	3 (6.1)	

Abbreviations: ASA, American Society of Anesthesiologists; CCI, Charlson comorbidity index; COPD, chronic obstructive pulmonary disease;  *median (IQR)*, median (interquartile range); ^1^ according to the Charlson comorbidity index

### Preoperative assessment and indication for surgery

Clinical presentation was found to be largely comparable between non-octogenarians and octogenarians. The mean symptom duration did not differ significantly between groups (*p* = 0.14). Median pain intensity, as measured by VAS, tended to be lower in octogenarians, reaching borderline statistical significance (*p* = 0.05). The distribution of the modified Alvarado score was similar between the two groups (*p* = 0.48), with most patients having scores “compatible with appendicitis”. Classical clinical signs, including the Blumberg, Lanz, and McBurney signs, were observed at comparable rates in both groups (all *p* ≥ 0.47). Laboratory findings showed no significant difference in leukocyte counts (*p* = 0.95), though CRP levels were significantly higher in octogenarians (*p* < 0.001). The indication for surgery was consistent across both groups, with most patients undergoing surgery immediately upon admission (*p* = 0.90). For more detailed results, refer to Table 3.

**Table 3:** Preoperative assessment and indication for surgery

Variable	No. (%)		*P* value
	Non-Octogenarians (age 18–79)	Octogenarians (age ≥ 80)	
Duration of symptoms, h, *median (IQR)*	24 (36)	24 (36)	0.14
Pain, VAS, *median (IQR)*	2 (5)	0 (4)	**0.05**
Modified Alvarado score (MASS)			
1–3 (unlikely)	370 (24.1)	7 (15.6)	0.48
4–6 (compatible)	664 (43.3)	29 (64.4)	
7–8 (probable)	278 (18.1)	8 (17.8)	
9 (very probable)	65 (4.2)	1 (2.2)	
Blumberg Sign	628 (46.2)	20 (46.5)	0.97
Lanz Sign	1052 (74.3)	31 (72.1)	0.74
McBurney Sign	1355 (90.2)	40 (86.9)	0.47
Leukocyte count, /nl, *median (IQR)*	12.8 (5.9)	12.8 (5.0)	0.95
CRP, mg/l, *median (IQR)*	26.7 (72.2)	83 (125.6)	**< 0.001**
Indication for surgery			
After inpatient observation	10 (20.4)	301 (19.7)	0.90
Immediately on admission	39 (79.6)	1230 (80.3)	

Abbreviations: CRP, C - reactive protein; MASS, modified Alvarado scoring system; *median (IQR)*, median (interquartile range); VAS, visual analog pain scale

### Surgical management

Significant differences in surgical management were observed between non-octogenarians and octogenarians. Although a simple appendectomy was the most common procedure in both groups, it was performed less frequently in octogenarians (83.7% vs. 94.2%). Octogenarians more often required extended resections, including appendectomy with cecal resection (10.2% vs. 4.0%), ileocecal resection (4.1% vs. 1.5%), and right hemicolectomy (2.0% vs. 0.3%; overall *p* = 0.01). The surgical approach differed significantly between the two groups (overall *p* < 0.001); laparoscopic surgery was less common in octogenarians with higher rates of open procedures (14.3% vs. 2.7%; *p* < 0.001) and conversion to open surgery (12.2% vs. 3.5%; *p* < 0.001). The median operating time did not differ significantly (60 min vs. 50 min; *p* = 0.09).

Ostomy creation was more frequent in octogenarians (2.1% vs. 0.2%; *p* = 0.01), as was the need for intraoperative placement of drainage (73.5% vs. 33.9%; *p* < 0.001). The overall rate of intraoperative complications was similar between the two groups (4.0% vs. 2.4%; *p* = 0.44). While bleeding and visceral injuries did not differ significantly, other injuries (e.g., spleen or ureter) occurred more frequently in octogenarians (4.3% vs. 0.2%; *p* < 0.001). Detailed results are provided in Table 4.

**Table 4:** Surgical management

Variable	No. (%)		*P* value
	Non-Octogenarians (age 18–79)	Octogenarians (age ≥ 80)	
Resection			
Appendectomy	1444 (94.2)	41 (83.7)	**0.01**
Appendectomy with cecal resection	61 (4.0)	5 (10.2)	
Ileocecal resection	23 (1.5)	2 (4.1)	
Right hemicolectomy	5 (0.3)	1 (2.0)	
Incision			
Laparoscopic	1438 (93.8)	36 (73.5)	**< 0.001**
Open	42 (2.7)	7 (14.3)	
Conversion	53 (3.5)	6 (12.2)	
Operating time, minutes, *median (IQR)*	50 (30)	60 (32)	0.09
Ostomy	3 (0.2)	1 (2.1)	**0.01**
Drainage	519 (33.9)	36 (73.5)	**< 0.001**
Intraoperative Complications (overall)	36 (2.4)	2 (4.0)	0.44
Bleeding	26 (1.8)	0 (0)	0.35
Visceral injury	7 (0.5)	1 (2.2)	0.13
Other injury (e.g. Spleen, Ureter)	3 (0.2)	2 (4.3)	**< 0.001**

Abbreviations: *median (IQR)*, median (interquartile range)

### Intraoperative findings / severity of appendicitis

Intraoperative findings and the (histopathological) severity of appendicitis differed significantly between age groups. Uncomplicated appendicitis was less prevalent in octogenarians, while complicated appendicitis was more prevalent in this age group (63.3% vs. 28.1%; *p* < 0.001). Octogenarians also exhibited significantly higher rates of perforation (59.2% vs. 23.0%; *p* < 0.001), peritonitis (65.3% vs. 39.7%; *p* < 0.001), and abscess formation (34.7% vs. 12.3%; *p* < 0.001). The distribution of the Mannheim Peritonitis Index differed significantly between groups (*p* < 0.001).

While the majority of patients in both groups were classified as low risk (MPI ≤ 20), a higher proportion of octogenarians were in the high-risk category (MPI ≥ 30: 3.2% vs. 1.3%). There were also significant differences in histopathological findings between the two groups (*p* = 0.03). Octogenarians showed lower rates of catarrhal and phlegmonous appendicitis, as well as higher proportions of advanced inflammatory stages. These included ulcero-phlegmonous (61.2% vs. 51.1%) and gangrenous appendicitis (24.5% vs. 16.1%). The rate of negative appendectomy was comparable (8.2% vs. 6.2%). See Table 5 for detailed results.

**Table 5:** Intraoperative findings / severity of appendicitis

Variable	No. (%)		*P* Value
	Non-Octogenarians (age 18–79)	Octogenarians (age ≥ 80)	
Uncomplicated appendicitis^a^	1102 (71.9)	18 (36.7)	**< 0.001**
Complicated appendicitis^b^	431 (28.1)	31 (63.3)	
Perforation	353 (23.0)	29 (59.2)	**< 0.001**
Peritonitis	608 (39.7)	32 (65.3)	**< 0.001**
Mannheim Peritonitis Index (MPI)			
MPI ≤ 20 (low risk)	544 (90.5)	28 (90.3)	**< 0.001**
MPI 21–29 (intermediate risk)	49 (8.2)	2 (6.4)	
MPI ≥ 30 (high risk)	8 (1.3)	1 (3.2)	
Abscess	188 (12.3)	17 (34.7)	**< 0.001**
Histopathology results			
No inflammation (neg. appendectomy)	95 (6.2)	4 (8.2)	**0.03**
Catarrhal	228 (14.9)	2 (4.1)	
Phlegmonous	179 (11.7)	1 (2.0)	
Ulcero-phlegmonous	784 (51.1)	30 (61.2)	
Gangrenous	247 (16.1)	12 (24.5)	

Abbreviations: ^a^phlegmonous appendicitis without perforation, abscess or peritonitis;

^b^gangrenous appendicitis or periappendicitis, peritonitis, perforation or abscess

### Postoperative management and outcome

Postoperative antimicrobial therapy (PAT) was administered more frequently in octogenarians than in non-octogenarians (79.6% vs. 41.6%; *p* < 0.001) and for a significantly longer duration (*p* < 0.001). Overall postoperative complications were more prevalent in octogenarians (*p* < 0.001), with fewer patients experiencing an uncomplicated postoperative course (53.0% vs. 87.1%). There were higher rates of both minor (Clavien-Dindo grade I–II: 34.7% vs. 8.9%) and major (grade III–V: 12.3% vs. 4.0%) complications and an increased mortality (2% vs. 0.1%; *p* = 0.002).

Octogenarians required intensive care unit (ICU) admission significantly more often (32.7% vs. 3.5%; *p* < 0.001). Although the median ICU stay was brief in both groups, it was marginally longer in octogenarians (0 ± 1 days vs. 0 ± 0 days; *p* = 0.04). Octogenarians had a significantly longer length of stay than non-octogenarians.

(7 ± 6 days vs. 3 ± 2 days; *p* < 0.001). A detailed summary of the results is presented in Table 6.

**Table 6:** Postoperative management and outcome

Variable	No. (%)		*P* Value
	Non-Octogenarians (age 18–79)	Octogenarians (age ≥ 80)	
PAT	638 (41.6)	39 (79.6)	**< 0.001**
Duration of PAT, d, *median (IQR)*	5 (4)	7 (5)	**< 0.001**
Postoperative complications^a^			
None	1335 (87.1)	26 (53.0)	**< 0.001**
Grade I – II	136 (8.9)	17 (34.7)	
Grade III – V	61 (4.0)	6 (12.3)	
Mortality	2 (0.1)	1 (2)	**0.002**
ICU	54 (3.5)	16 (32.7)	**< 0.001**
ICU stay, d, *median (IQR)*	0 (0)	0 (1)	**0.04**
LOS, d, *median (IQR)*	3 (2)	7 (6)	**< 0.001**

Abbreviations: ICU, intensive-care-unit; LOS, length-of-stay; *median (IQR)*, median (interquartile range); PAT, postoperative antimicrobial therapy; ^a^according to Clavien-Dindo

### Outcomes after 1:1 propensity score matching

The baseline characteristics of the matched cohort are summarized in Supplementary Table 1. Propensity score matching substantially improved covariate balance compared with the unmatched cohort (Supplementary Fig. 1). Most standardized mean differences (SMD) were below the commonly accepted threshold of 0.2, with only modest residual imbalance observed for ASA score (SMD − 0.260), oral anticoagulation (SMD 0.239), and immunosuppression (SMD 0.213).

ICU admission did not differ significantly between octogenarians and controls (30.6% vs. 16.7%; *p* = 0.267). Likewise, the proportion of patients receiving PAT was comparable between groups (77.8% vs. 69.4%; *p* = 0.593). The proportion of patients without postoperative complications (Clavien–Dindo grade 0) was similar in octogenarians and controls (47.2% vs. 52.8%; *p* = 0.814). No significant differences were observed for minor complications (grades I–II; 41.7% vs. 30.6%;

*p* = 0.462) or major complications (grade III–V; 11.1% vs. 16.7%; *p* = 0.735), or the incidence of any postoperative complication (grade > 0; 52.8% vs. 47.2%; *p* = 0.814). The length of hospital stay was comparable, at a median of 7.0 days (IQR 4.0–10.2) in octogenarians and 6.0 days (IQR 4.0–10.0) in matched controls (*p* = 0.850). These findings are summarized in Supplementary Table 2.

### Outcomes after 1:2 propensity score matching

Sensitivity analysis using 1:2 propensity score matching included 36 octogenarians and 66 controls. Baseline covariate balance remained acceptable overall (Supplementary Fig. 2), although residual imbalance was slightly greater than in the 1:1 matched cohort, particularly for oral anticoagulation (SMD 0.269),

ASA score (SMD − 0.238), and immunosuppression (SMD 0.160).

As in the primary analysis, ICU admission was not significantly different between octogenarians and matched controls (30.6% vs. 19.7%, *p* = 0.231). PAT was administered to 77.8% of octogenarians and 66.7% of controls (*p* = 0.342). The proportion of patients without postoperative complications (Clavien–Dindo grade 0) was similar between groups (47.2% vs. 54.5%; *p* = 0.617). Likewise, neither minor complications (grade I–II; 41.7% vs. 30.3%; *p* = 0.349) nor major complications (grade III-V; 11.1% vs. 15.2%; *p* = 0.765) differed significantly. The overall incidence of postoperative complications (Clavien–Dindo grade > 0) was comparable (52.8% vs. 45.5%; *p* = 0.617), and the median length of hospital stay did not differ significantly between octogenarians and controls (median 7.0 days [IQR 4.0–10.2] vs. 6.0 days [IQR 4.0–9.0]; *p* = 0.542). These results are summarized in Supplementary.

### Consistency across matching strategies

The overall pattern of postoperative outcomes was consistent across both matching approaches. Octogenarians showed numerically higher rates of ICU admission and PAT in both matched cohorts; however, none of these differences reached statistical significance. Similarly, postoperative complications, including overall, minor and major complications, as well as LOS were comparable between octogenarians and matched controls irrespective of the matching ratio. The 1:1 matching strategy achieved slightly better covariate balance than the 1:2 approach and therefore served as the primary analysis, whereas the 1:2 matched cohort confirmed the robustness of the findings in a sensitivity analysis. Supplementary Figs. 3 and 4 provide a direct comparison of key postoperative outcomes.

### Attrition analysis of unmatched octogenarians

In order to assess potential selection bias introduced by propensity score matching, baseline characteristics of matched and unmatched octogenarians were compared (see supplementary Table 3). No statistically significant differences were observed between the two groups regarding demographic characteristics, comorbidity burden (ASA score and Charlson Comorbidity Index), oral anticoagulation, COPD, immunosuppression, diabetes mellitus, obesity, sex, or the proportion of complicated appendicitis (all *p* > 0.05). The findings of this study suggest that the matched cohort reflects key characteristics of the overall octogenarian population. Exclusion from the matched analysis, mainly due to the lack of a suitable control (*n* = 9) or missing matching covariates (*n* = 4), was not associated with clinically relevant baseline differences.

## Discussion

This multicenter, retrospective cohort study revealed marked age-related variations in disease severity, surgical management, and postoperative outcomes in patients undergoing surgery for acute appendicitis. Octogenarians constituted a small but distinct subgroup characterized by a higher comorbidity burden, more advanced appendiceal disease at presentation, and worse postoperative outcomes compared to non-octogenarians.

A key finding of this study is the marked shift toward complicated appendicitis in octogenarians. While uncomplicated appendicitis was more common in younger patients, nearly two-thirds of octogenarians presented with complicated disease. This shift is reflected by significantly higher rates of perforation, peritonitis, abscess formation, and advanced histopathological stages, such as gangrenous or ulcero-phlegmonous appendicitis. These results align with previous reports indicating that appendicitis in the elderly often tends to progress more aggressively and is often diagnosed at a later stage [21, 22]. The presence of atypical or attenuated clinical symptoms, evidenced by lower pain scores in octogenarians despite comparable symptom duration, may contribute to diagnostic delays and disease progression in this population [23].

Interestingly, preoperative clinical assessments and tools, such as the modified Alvarado score and classical physical examination findings, did not differ significantly among age groups. Notably, the inflammatory marker CRP was significantly elevated in octogenarians, potentially serving as a reliable indicator of disease severity in this age group. These results underscore the diagnostic challenges of appendicitis in the elderly and emphasize the importance of maintaining a high level of suspicion and making liberal use of imaging and laboratory parameters.

The increased severity of intraoperative findings in octogenarians resulted in more complex surgical management. Elderly patients more frequently required extended resections, open surgery, conversion from laparoscopy, ostomy creation, and intraoperative drainage. These differences likely reflect advanced local disease and the need for tailored surgical strategies given age-related comorbidities.

Despite these differences, the overall operating time and rate of intraoperative complications were comparable, suggesting that surgery can be safely performed on octogenarians when tailored appropriately. However, rare but severe injuries occurred more frequently in this group.

Postoperative outcomes were significantly worse in octogenarians. They experienced higher rates of minor and major complications, increased mortality, more frequent ICU admissions, longer courses of postoperative antimicrobial therapy, and prolonged hospital stays. These findings are likely multifactorial, driven by advanced disease stage, reduced physiological reserve, and higher baseline comorbidity and ASA scores. Importantly, the substantially increased need for ICU care highlights the relevance of perioperative risk stratification and resource planning when treating elderly patients with appendicitis.

The propensity score-matched analyses provide important additional context for interpreting our findings. After adjustment for baseline differences in comorbidity burden and perioperative risk, as well as disease severity (complicated appendicitis), the previously observed disparities in postoperative outcomes between octogenarians and younger patients were no longer statistically significant. After Matching, baseline covariate balance improved substantially, although small residual imbalances remained for ASA score and oral anticoagulation. In both the 1:1 and 1:2 matched cohorts, no statistically significant differences were observed regarding ICU admission, postoperative complications, or LOS between octogenarians and matched controls.

These findings suggest that the poorer postoperative outcomes observed in the unmatched cohort were largely explained by differences in the baseline comorbidity burden and disease severity at presentation rather than chronological age alone. In summary, younger patients with a similar spectrum of comorbidities and comparable appendicitis severity (complicated appendicitis) seem to have outcomes comparable to octogenarians.

This indicates that octogenarians are a high-risk subgroup, primarily due to their age-related comorbidities, rather than their chronological age. Consequently, the vulnerabilities typically attributed to octogenarians should also be considered in younger but similarly comorbid patients.

### Limitations

This study has several strengths, including its large overall sample size and a multicenter design across different hospital care levels. It also has a comprehensive assessment of clinical, intraoperative, histopathological, and postoperative variables using standardized definitions. Nevertheless, some limitations must be acknowledged. Although the overall sample size was large (1,582 patients), only a small percentage (49 patients, 3.1%) were octogenarians, which may limit statistical power for subgroup analyses. Also, only patients who underwent surgery were included, precluding conclusions about non-operative management strategies for the elderly. The retrospective design carries an inherent risk of selection bias and missing data.

Propensity score matching was performed to improve comparability between octogenarians and younger patients by balancing important baseline characteristics, including comorbidity burden and appendicitis severity. Although covariate balance improved substantially after matching, small residual imbalances remained for ASA score and oral anticoagulation in the matched cohorts, limiting causal inference.

Furthermore, only 36 of the 49 octogenarians could be included in the matched analysis. Although an attrition analysis demonstrated no statistically significant baseline differences between matched and unmatched octogenarians, exclusion of patients without suitable matches or with incomplete covariate data may nevertheless have resulted in a selection bias. Consequently, the matched analyses may underestimate postoperative risks among the most vulnerable older patients, particularly those who may not have undergone surgery or who could not be adequately matched.

An important clinical implication of these findings concerns the role of non-operative management (NOM) in Octogenarians. The markedly higher prevalence of complicated appendicitis in this age group emphasizes the need for careful assessment of disease severity before considering NOM, as complicated appendicitis usually requires operative management. However, these findings should not be interpreted as advocating routine operative management solely on the basis of chronological age. Rather, NOM should be reserved for carefully selected patients with uncomplicated appendicitis after thorough clinical and radiological evaluation. This advisory also applies to younger patients with a comparable comorbidity burden.

Finally, the study period coincided partly with the COVID-19 pandemic. Pandemic-related changes in healthcare utilization, including delayed presentation or avoidance of hospital care, particularly among older adults, were not specifically evaluated and may have influenced appendicitis severity and treatment patterns.

## Conclusion

Octogenarians with acute appendicitis more frequently present with advanced disease and experience worse postoperative outcomes than younger patients in the unadjusted analysis, despite similar initial clinical presentations. However, after adjustment for baseline differences using propensity score matching, the postoperative outcomes became largely comparable. The risk for worse outcomes appears to be driven primarily by differences in age-related comorbidity burden rather than chronological age. This underscores the need for heightened diagnostic vigilance, accurate assessment of appendicitis severity, and tailored intervention and perioperative management strategies in elderly patients as well as in younger patients with a comparable spectrum of comorbidity burden. Given the high prevalence of complicated appendicitis among octogenarians, non-operative management should be considered only in carefully selected patients with uncomplicated disease. 

## Supplementary Information

Supplementary Material 1.

## Data Availability

No datasets were generated or analysed during the current study.
