# Supplementary material for: Does age matter? Acute appendicitis in octogenarians vs. younger patients - results of a German multicenter analysis
Source: Langenbecks Arch Surg. 2026 Sep 8;411(1):230. doi: 10.1007/s00423-026-04202-3 (PMC13562195; doi:10.1007/s00423-026-04202-3)
Supplement: Supplementary file 1 [file 423_2026_4202_MOESM1_ESM.docx]

**Supplementary Table 1: Baseline characteristics after propensity score matching using 1:1 and 1:2 matching approaches**

| Variable | Ratio | Controls | Octogenarians | SMD |
| --- | --- | --- | --- | --- |
| n (matched sample) | **1:1** | 36 | 36 |  |
| Female Sex |  | 15 (41.7%) | 16 (44.4%) | 0.057 |
| Obesity |  | 3 (8.3%) | 4 (11.1%) | 0.094 |
| Diabetes mellitus |  | 5 (13.9%) | 4 (11.1%) | -0.094 |
| COPD |  | 2 (5.6%) | 3 (8.3%) | 0.094 |
| Oral anticoagulation |  | 8 (22.2%) | 12 (33.3%) | 0.239 |
| Immunosuppression |  | 1 (2.8%) | 3 (8.3%) | 0.213 |
| ASA score*, median [IQR]* |  | 3.0 [3.0–3.0] | 3.0 [2.0–3.0] | -0.260 |
| Charlson Comorbidity Index, *median [IQR]* |  | 2.0 [0.0–4.0] | 2.0 [0.0–5.0] | 0.020 |
| Complicated Appendicitis |  | 31 (86.1%) | 31 (86.1%) | 0.000 |
| n (matched sample) | **1:2** | 66 | 36 |  |
| Female Sex |  | 31 (47.0%) | 16 (44.4%) | 0.000 |
| Obesity |  | 6 (8.3%) | 4 (11.1%) | 0.094 |
| Diabetes mellitus |  | 8 (11.1%) | 4 (11.1%) | 0.000 |
| COPD |  | 4 (5.6%) | 3 (8.3%) | 0.094 |
| Oral anticoagulation |  | 15 (20.8%) | 12 (33.3%) | 0.269 |
| Immunosuppression |  | 3 (4.2%) | 3 (8.3%) | 0.160 |
| ASA score, *median [IQR]* |  | 3.0 [2.0–3.0] | 3.0 [2.0–3.0] | -0.238 |
| Charlson Comorbidity Index, *median [IQR]* |  | 1.0 [0.0–4.0] | 2.0 [0.0–5.0] | 0.030 |
| Complicated Appendicitis |  | 58 (87.9%) | 31 (86.1%) | -0.039 |

Abbreviations: *SMD*, standardized mean differences; *COPD*, chronic obstructive pulmonary disease; *ASA*, American Society of Anesthesiologists

Baseline demographic and clinical characteristics of octogenarians and propensity score matched controls are presented for both matching strategies. The 1:1 matched cohort represents the primary analysis, while the 1:2 matched cohort is shown as a sensitivity analysis. Categorical variables are reported as counts and percentages, and continuous variables as median with interquartile range [IQR]. Covariate balance between groups was assessed using standardized mean differences (SMD), with values <0.1 indicating optimal balance and values <0.2 considered acceptable. Both matching approaches substantially reduced baseline imbalance compared with the unmatched cohort and achieved excellent balance for most covariates, including complicated appendicitis. Small residual imbalances remained for ASA score, oral anticoagulation, and immunosuppression, with comparable overall covariate balance observed for the 1:1 and 1:2 matched cohort.

**Supplementary Table 2:**

**Postoperative Outcomes after Propensity Score Matching using 1:1 and 1:2 matching approaches**

| Variable | Ratio | Controls | Octogenarians | p-Value |
| --- | --- | --- | --- | --- |
| **n (matched sample)** | **1:1** | 36 | 36 |  |
| ICU admission |  | 6 (16.7%) | 11 (30.6%) | 0.267 |
| Postop antimicrobial therapy (PAT) |  | 25 (69.4%) | 28 (77.8%) | 0.593 |
| Postop complications: None (Dindo = 0) |  | 19 (52.8%) | 17 (47.2%) | 0.814 |
| Postop complications: Grade I–II (Dindo 1–2) |  | 11 (30.6%) | 15 (41.7%) | 0.462 |
| Postop complications: Grade III–V  (Dindo 3–5) |  | 6 (16.7%) | 4 (11.1%) | 0.735 |
| Any postoperative complication  (Dindo > 0) |  | 17 (47.2%) | 19 (52.8%) | 0.814 |
| Major complications (Dindo ≥ 3) |  | 6 (16.7%) | 4 (11.1%) | 0.735 |
| Length of stay (LOS), *median [IQR]* |  | 6.0 [4.0–10.0] | 7.0 [4.0–10.2] | 0.850 |
| n (matched sample) | **1:2** | 66 | 36 |  |
| ICU admission |  | 13 (19.7%) | 11 (30.6%) | 0.231 |
| Postop antimicrobial therapy (PAT) |  | 44 (66.7%) | 28 (77.8%) | 0.342 |
| Postop complications: None (Dindo = 0) |  | 36 (54.5%) | 17 (47.2%) | 0.617 |
| Postop complications: Grade I–II (Dindo 1–2) |  | 20 (30.3%) | 15 (41.7%) | 0.349 |
| Postop complications: Grade III–V  (Dindo 3–5) |  | 10 (15.2%) | 4 (11.1%) | 0.765 |
| Any postoperative complication  (Dindo > 0) |  | 30 (45.5%) | 19 (52.8%) | 0.617 |
| Major complications (Dindo ≥ 3) |  | 10 (15.2%) | 4 (11.1%) | 0.765 |
| Length of stay (LOS), *median [IQR]* |  | 6.0 [4.0–9.0] | 7.0 [4.0–10.2] | 0.542 |

Abbreviations: *PAT*, postoperative antimicrobial therapy; *LOS,* length-of-stay;
*ICU*, intensive-care-unit; *IQR,* interquartile range

Outcomes are presented for octogenarians and matched controls after propensity score matching. The 1:1 matched cohort represents the primary analysis, whereas the 1:2 matched cohort is provided as a sensitivity analysis. Categorical variables are reported as counts with percentages and continuous variables as median with interquartile range [IQR]. P-values were calculated using Pearson’s χ² test or Fisher's exact test for categorical variables, as appropriate according to cell frequencies, and the Wilcoxon rank-sum test for length of stay (LOS). Across both matching strategies, postoperative outcomes – including ICU admission, postoperative antimicrobial therapy, postoperative complications according to Clavien-Dindo classification, overall complication rate, major complications and LOS – did not differ significantly between octogenarians and matched controls (all p > 0.05).

**Supplementary Table 3: Attrition analysis of matched and unmatched Octogenarians in the propensity score matching analysis**

| Variable | Matched Octogenarians | Unmatched Octogenarians | p-Value |
| --- | --- | --- | --- |
| n | 36 | 13 |  |
| Female Sex | 16 (44.0%) | 4 (31.0%) | 0.390 |
| Obesity (BMI >30 kg/m²) | 4 (11.0%) | 0 (0.0%) | 0.569 |
| - Unknown | 0 (0.0%) | 4 (30.8%) |  |
| Diabetes mellitus | 4 (11.0%) | 0 (0.0%) | 0.562 |
| COPD | 3 (8.3%) | 2 (15.0%) | 0.598 |
| Oral anticoagulation | 12 (33.3%) | 4 (31.0%) | 0.999 |
| Immunosuppression | 3 (8.3%) | 1 (7.7%) | 0.999 |
| ASA score |  |  | 0.622 |
| - 1-2 | 14 (38.8%) | 2 (15.4%) |  |
| - 3 | 20 (56.0%) | 7 (53.8%) |  |
| - 4 | 2 (5.6%) | 1 (7.7%) |  |
| - Unknown | 0 (0.0%) | 3 (23.1%) |  |
| Charlson Comorbidity Index |  |  | 0.215 |
| - 0-2 | 21 (58.3%) | 5 (38.5%) |  |
| - 3-4 | 4 (11.1%) | 1 (7.7%) |  |
| - ≥ 5 | 11 (30.6%) | 4 (30.8%) |  |
| - Unknown | 0 (0%) | 3 (23.1%) |  |
| Complicated Appendicitis | 31 (86.0%) | 10 (77.0%) | 0.422 |

Abbreviations: *COPD*, chronic obstructive pulmonary disease; *ASA*, American Society of Anesthesiologists

Baseline demographic and clinical characteristics of octogenarians successfully matched to younger controls were compared with those octogenarians excluded from the matched cohort because no suitable control could be identified within the predefined matching criteria (9 cases) or due to missing values in the key covariates (4 cases). Categorical variables are presented as counts and percentages. No statistically significant differences were observed between matched and unmatched octogenarians regarding age-adjusted comorbidity burden (Charlson Comorbidity Index), ASA score, oral anticoagulation, chronic obstructive pulmonary disease, immunosuppression, diabetes mellitus, sex, obesity, or appendicitis severity (all p > 0.5), indicating that exclusion from the matched cohort was not associated with clinically relevant baseline differences.

**Supplementary Figure 1:**

**Covariate balance before and after 1:1 propensity score matching**

Abbreviations: *ASA*, American Society of Anesthesiologists, *COPD*, chronic obstructive pulmonary disease; *Diabetes*, diabetes mellitus

This Love plot illustrates the distribution of absolute standardized mean differences (SMDs) for all baseline covariates before and after 1:1 nearest-neighbor propensity score matching. Prior to matching, substantial baseline imbalance was observed between octogenarians and controls across several demographic and clinical characteristics, particularly ASA score, Charlson Comorbidity Index, oral anticoagulation, and complicated appendicitis. After matching, covariate balance improved markedly, with most variables achieving acceptable balance (|SMD| < 0.2), and several reaching optimal balance (|SMD| < 0.1). Complete balance (SMD = 0) was achieved for complicated appendicitis, whereas small residual imbalance remained for ASA score, oral anticoagulation, and immunosuppression. Overall, the 1:1 propensity score matching substantially reduced baseline differences and improved cohort comparability.

**Supplementary Figure 2:**

**Covariate balance before and after 1:2 propensity score matching**

Abbreviations: *ASA*, American Society of Anesthesiologists, *COPD*, chronic obstructive pulmonary disease; *Diabetes*, diabetes mellitus

This Love plot illustrates the distribution of absolute standardized mean differences (SMDs) for all baseline covariates before and after 1:2 nearest-neighbor propensity score matching (one octogenarian matched to two controls). Before matching, substantial baseline imbalance was observed across several demographic and clinical characteristics, particularly ASA score, Charlson Comorbidity Index, oral anticoagulation, and complicated appendicitis. After matching, covariate balance improved markedly, with most variables achieving acceptable balance (|SMD| < 0.2) and several reaching excellent balance (|SMD| < 0.1). Complete balance (SMD = 0) was achieved for sex and diabetes mellitus, while only small residual imbalance remained for ASA score, oral anticoagulation, and immunosuppression. Compared with the 1:1 matching approach, the 1:2 strategy retained a larger control sample while achieving a similarly favorable overall balance, although residual imbalance for ASA score and oral anticoagulation was slightly more pronounced.

**Supplementary Figure 3:**

**Comparison of key postoperative outcomes after 1:1 Propensity Score Matching**

Abbreviations: *ICU*, intensive care unit; *PAT*, postoperative antimicrobial therapy; *LOS*, length of hospital stay.

These figures summarize the distribution of major postoperative outcomes in octogenarians and propensity score-matched controls. Panel A displays the proportions of patients requiring intensive care unit (ICU) admission, postoperative antimicrobial therapy (PAT), and experiencing any postoperative complication (Clavien–Dindo grade >0), whereas Panel B illustrates the distribution of length of hospital stay (LOS). Across the matched cohort, octogenarians showed numerically higher rates of ICU admission (30.6% vs. 16.7%) and postoperative antimicrobial therapy (77.8% vs. 69.4%) than matched controls, while the overall rate of postoperative complications (52.8% vs. 47.2%) and LOS (median 7.0 vs. 6.0 days) were comparable between groups. None of the observed differences reached statistical significance. P-values shown in the figure correspond to statistical comparisons using Fisher’s exact test for ICU admission, Pearson’s chi-squared test for PAT and overall postoperative complications, and the Wilcoxon rank-sum test for LOS.
**Supplementary Figure 4:**

**Comparison of key postoperative outcomes after 1:2 Propensity Score Matching**

Abbreviations: *ICU*, intensive care unit; *PAT*, postoperative antimicrobial therapy; *LOS*, length of hospital stay.

These figures summarize the distribution of major postoperative outcomes in octogenarians and propensity score-matched controls following 1:2 matching. Panel A displays the proportions of patients requiring intensive care unit (ICU) admission, postoperative antimicrobial therapy (PAT), and experiencing any postoperative complication (Clavien–Dindo grade >0), whereas Panel B illustrates the distribution of length of hospital stay (LOS). Across the matched cohort, octogenarians showed numerically higher rates of ICU admission (30.6% vs. 19.7%) and postoperative antimicrobial therapy (77.8% vs. 66.7%) than matched controls, while the overall rate of postoperative complications (52.8% vs. 45.5%) and LOS (median 7.0 vs. 6.0 days) were comparable between groups. None of the observed differences reached statistical significance. P-values shown in the figure correspond to statistical comparisons using Fisher’s exact test for ICU admission, Pearson’s chi-squared test for PAT and overall postoperative complications, and the Wilcoxon rank-sum test for LOS.
